# Supplementary material for: Anomaly detection in smart power grids with graph-regularized MS-SVDD: a multimodal subspace learning approach
Source: Sci Rep. 2026 Apr 24;16:18896. doi: 10.1038/s41598-026-46799-x (PMC13276183; doi:10.1038/s41598-026-46799-x)
Supplement: Supplementary file 1 — Supplementary Information. [file 41598_2026_46799_MOESM1_ESM.pdf]

Anomaly Detection in Smart Power Grids with Graph-Regularized MS-SVDD: Supplementary Material

Thomas Debelle[1], Fahad Sohrab*[2], Pekka Abrahamsson[2], and Moncef Gabbouj[2]

[1]Technical University of Darmstadt, Karolinenplatz 5, 64289 Darmstadt, Germany
[2]Faculty of Information Technology and Communication Sciences, Tampere University, FI-33720 Tampere, Finland

This document contains supplementary material for the proposed Graph-Regularized Multi-modal Subspace Support Vector Data Description (MS-SVDD). Section 1 provides a chart describing the preprocessing steps to create the reliability dataset. In Section 2, we report all the experimental results for MS-SVDD and CCS-SVDD, as well as the best hyperparameters found.

*fahad.sohrab@tuni.fi

1 Preprocessing of the PSML dataset for reliability evaluation

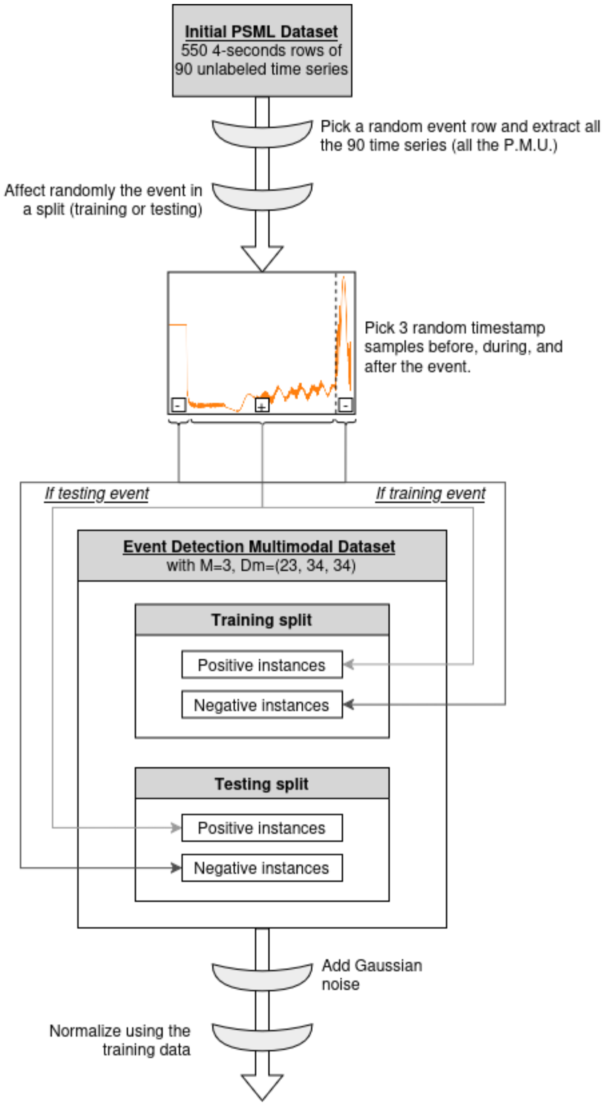

Figure 1: Preprocessing of PSML dataset for reliability evaluation.

2 Full results of the experiments

Strat	NPT	Noise	σ	C	β	η	d	Acc	TPR	TNR	Pre	GM	train time	test time
AND	0	0	0	0.02	10.0	0.1	10	0.47	0.85	0.09	0.48	0.28	39.572	0.010
AND	0	1	0	0.02	1.0	0.1	10	0.48	0.86	0.11	0.49	0.31	39.607	0.010
AND	1	0	0.01	0.01	100.0	0.1	10	0.67	0.85	0.49	0.63	0.65	55.580	0.652
AND	1	1	0.01	0.01	100.0	0.1	10	0.53	0.84	0.23	0.52	0.44	60.956	1.423
OR	0	0	0	0.02	10.0	0.1	10	0.50	1.00	0.01	0.50	0.08	38.641	0.011
OR	0	1	0	0.02	0.01	0.1	15	0.50	1.00	0.00	0.50	0.00	40.117	0.014
OR	1	0	0.01	0.01	100.0	0.1	10	0.50	1.00	0.00	0.50	0.00	55.205	0.468
OR	1	1	0.01	0.01	100.0	0.1	10	0.50	0.99	0.00	0.50	0.00	61.283	0.644
UNI_0	0	0	0	0.02	10.0	0.1	10	0.47	0.85	0.09	0.48	0.28	39.722	0.011
UNI_0	0	1	0	0.02	1.0	0.1	10	0.48	0.86	0.11	0.49	0.31	39.032	0.011
UNI_0	1	0	0.01	0.01	100.0	0.1	10	0.67	0.85	0.49	0.63	0.65	55.566	0.487
UNI_0	1	1	0.01	0.01	100.0	0.1	10	0.53	0.84	0.23	0.52	0.44	60.874	0.673
UNI_1	0	0	0	0.02	10.0	0.1	10	0.49	0.90	0.09	0.50	0.29	47.158	0.011
UNI_1	0	1	0	0.02	1.0	0.1	10	0.52	0.92	0.11	0.51	0.32	38.951	0.011
UNI_1	1	0	0.01	0.01	100.0	0.1	10	0.67	0.98	0.36	0.60	0.59	56.016	0.485
UNI_1	1	1	0.01	0.01	100.0	0.1	10	0.56	0.96	0.16	0.53	0.39	61.421	0.718
UNI_2	0	0	0	0.02	10.0	0.1	10	0.49	0.90	0.09	0.50	0.29	47.140	0.011
UNI_2	0	1	0	0.02	1.0	0.1	10	0.52	0.92	0.11	0.51	0.32	39.967	0.011
UNI_2	1	0	0.01	0.01	100.0	0.1	10	0.67	0.98	0.36	0.60	0.59	61.586	0.495
UNI_2	1	1	0.01	0.01	100.0	0.1	10	0.56	0.96	0.16	0.53	0.39	61.890	0.72

Table 1: Full results for CCS-SVDD experiments.

Reg.	Strat	NPT	Noise	σ	k	C	β	η	d	Acc	TPR	TNR	Pre	GM	train time	test time
0	AND	0	0	0	0	0.01	0.001	0.1	5	0.40	0.71	0.10	0.44	0.26	0.177	0.004
0	AND	0	1	0	0	0.01	0.01	0.1	2	0.41	0.71	0.11	0.44	0.28	0.124	0.003
0	AND	1	0	0.01	0	0.01	1.0	0.1	5	0.72	0.85	0.58	0.67	0.70	2.753	0.509
0	AND	1	1	0.01	0	0.01	10000.0	0.1	4	0.67	0.88	0.46	0.62	0.64	3.477	0.597
0	OR	0	0	0	0	0.2	1.0	0.1	10	0.50	1.00	0.00	0.50	0.00	0.198	0.006
0	OR	0	1	0	0	0.5	0.001	0.1	4	0.50	1.00	0.00	0.50	0.00	0.312	0.006
0	OR	1	0	0.01	0	0.01	10000.0	0.1	4	0.61	0.99	0.23	0.56	0.47	4.442	0.476
0	OR	1	1	0.01	0	0.01	10000.0	0.1	4	0.53	0.99	0.07	0.52	0.27	2.937	0.583
0	UNI_0	0	0	0	0	0.01	0.001	0.1	5	0.40	0.71	0.10	0.44	0.26	0.171	0.004
0	UNI_0	0	1	0	0	0.01	0.01	0.1	2	0.41	0.71	0.11	0.44	0.28	0.200	0.005
0	UNI_0	1	0	0.01	0	0.01	1.0	0.1	5	0.72	0.85	0.58	0.67	0.70	2.848	0.533
0	UNI_0	1	1	0.01	0	0.01	10000.0	0.1	4	0.67	0.88	0.46	0.62	0.64	3.399	0.582
0	UNI_1	0	0	0	0	0.01	0.001	0.1	5	0.48	0.88	0.07	0.49	0.25	0.249	0.008
0	UNI_1	0	1	0	0	0.01	0.001	0.1	5	0.46	0.87	0.06	0.48	0.23	0.175	0.004
0	UNI_1	1	0	0.01	0	0.01	10000.0	0.1	4	0.76	0.93	0.58	0.69	0.74	2.303	0.476
0	UNI_1	1	1	0.01	0	0.01	10000.0	0.1	4	0.69	0.95	0.43	0.62	0.64	2.521	0.535
0	UNI_2	0	0	0	0	0.01	0.001	0.1	5	0.48	0.88	0.07	0.49	0.25	0.188	0.008
0	UNI_2	0	1	0	0	0.01	0.001	0.1	5	0.46	0.87	0.06	0.48	0.23	0.240	0.008
0	UNI_2	1	0	0.01	0	0.01	10000.0	0.1	4	0.76	0.93	0.58	0.69	0.74	4.470	0.538
0	UNI_2	1	1	0.01	0	0.01	10000.0	0.1	4	0.69	0.95	0.43	0.62	0.64	5.204	0.652
1	AND	0	0	0	0	0.01	0.01	0.1	2	0.40	0.70	0.09	0.44	0.25	0.155	0.003
1	AND	0	1	0	0	0.01	10.0	0.1	5	0.42	0.72	0.13	0.45	0.30	0.374	0.008
1	AND	1	0	0.01	0	0.01	10000.0	0.1	4	0.72	0.85	0.60	0.68	0.71	2.461	0.518
1	AND	1	1	0.01	0	0.01	1.0	0.1	5	0.65	0.84	0.46	0.61	0.62	5.873	0.451
1	OR	0	0	0	0	0.01	10.0	0.1	5	0.50	1.00	0.01	0.50	0.08	0.445	0.010
1	OR	0	1	0	0	0.01	10.0	0.1	5	0.50	1.00	0.00	0.50	0.00	0.482	0.010
1	OR	1	0	0.01	0	0.01	10000.0	0.1	4	0.60	0.99	0.20	0.55	0.45	4.590	0.320
1	OR	1	1	0.01	0	0.01	10000.0	0.1	4	0.54	0.99	0.09	0.52	0.29	3.313	1.046
1	UNI_0	0	0	0	0	0.01	0.01	0.1	2	0.40	0.70	0.09	0.44	0.25	0.378	0.007
1	UNI_0	0	1	0	0	0.01	10.0	0.1	5	0.42	0.72	0.13	0.45	0.30	0.420	0.010
1	UNI_0	1	0	0.01	0	0.01	10000.0	0.1	4	0.72	0.85	0.60	0.68	0.71	4.281	1.796
1	UNI_0	1	1	0.01	0	0.01	1.0	0.1	5	0.65	0.84	0.46	0.61	0.62	3.043	1.132
1	UNI_1	0	0	0	0	0.01	10.0	0.1	5	0.48	0.84	0.12	0.49	0.32	0.336	0.011
1	UNI_1	0	1	0	0	0.01	10.0	0.1	5	0.48	0.83	0.12	0.49	0.32	0.482	0.010

1	UNI$_1$	1	0	0.01	0	0.01	10000.0	0.1	4	0.76	0.93	0.58	0.69	0.74	2.868	0.385
1	UNI$_1$	1	1	0.01	0	0.01	10000.0	0.1	4	0.71	0.95	0.47	0.64	0.67	2.772	0.765
1	UNI$_2$	0	0	0	0	0.01	10.0	0.1	5	0.48	0.84	0.12	0.49	0.32	0.366	0.010
1	UNI$_2$	0	1	0	0	0.01	10.0	0.1	5	0.48	0.83	0.12	0.49	0.32	0.539	0.010
1	UNI$_2$	1	0	0.01	0	0.01	10000.0	0.1	4	0.76	0.93	0.58	0.69	0.74	3.934	0.370
1	UNI$_2$	1	1	0.01	0	0.01	10000.0	0.1	4	0.71	0.95	0.47	0.64	0.67	3.245	0.867
2	AND	0	0	0	0	0.01	100.0	0.1	3	0.39	0.68	0.10	0.43	0.26	0.224	0.008
2	AND	0	1	0	0	0.01	10.0	0.1	5	0.41	0.72	0.11	0.45	0.28	0.456	0.011
2	AND	1	0	0.01	0	0.01	1.0	0.1	5	0.77	0.86	0.67	0.72	0.76	3.494	0.380
2	AND	1	1	0.01	0	0.01	10000.0	0.1	4	0.67	0.9	0.45	0.62	0.63	4.528	0.773
2	OR	0	0	0	0	0.01	10.0	0.1	5	0.50	1.00	0.01	0.5	0.08	0.461	0.010
2	OR	0	1	0	0	0.5	0.001	0.1	4	0.50	1.00	0.00	0.5	0.00	0.401	0.012
2	OR	1	0	0.01	0	0.01	10000.0	0.1	4	0.59	0.99	0.18	0.55	0.43	4.960	0.394
2	OR	1	1	0.01	0	0.01	1.0	0.1	5	0.52	0.99	0.04	0.51	0.21	5.044	0.660
2	UNI$_0$	0	0	0	0	0.01	100.0	0.1	3	0.39	0.68	0.10	0.43	0.26	0.490	0.008
2	UNI$_0$	0	1	0	0	0.01	10.0	0.1	5	0.41	0.72	0.11	0.45	0.28	0.476	0.010
2	UNI$_0$	1	0	0.01	0	0.01	1.0	0.1	5	0.77	0.86	0.67	0.72	0.76	5.108	0.484
2	UNI$_0$	1	1	0.01	0	0.01	10000.0	0.1	4	0.67	0.90	0.45	0.62	0.63	4.862	0.685
2	UNI$_1$	0	0	0	0	0.01	100.0	0.1	3	0.47	0.84	0.10	0.48	0.29	0.403	0.010
2	UNI$_1$	0	1	0	0	0.01	10.0	0.1	5	0.47	0.87	0.06	0.48	0.23	0.476	0.010
2	UNI$_1$	1	0	0.01	0	0.01	1.0	0.1	5	0.80	0.95	0.65	0.73	0.79	5.856	0.424
2	UNI$_1$	1	1	0.01	0	0.01	10000.0	0.1	4	0.68	0.95	0.41	0.62	0.62	2.511	0.442
2	UNI$_2$	0	0	0	0	0.01	100.0	0.1	3	0.47	0.84	0.10	0.48	0.29	0.295	0.005
2	UNI$_2$	0	1	0	0	0.01	10.0	0.1	5	0.47	0.87	0.06	0.48	0.23	0.394	0.010
2	UNI$_2$	1	0	0.01	0	0.01	1.0	0.1	5	0.80	0.95	0.65	0.73	0.79	3.626	0.396
2	UNI$_2$	1	1	0.01	0	0.01	10000.0	0.1	4	0.68	0.95	0.41	0.62	0.62	2.866	0.719
3	AND	0	0	0	0	0.01	10000.0	0.1	3	0.39	0.70	0.09	0.43	0.24	0.418	0.008
3	AND	0	1	0	0	0.01	0.01	0.1	2	0.41	0.71	0.11	0.44	0.28	0.249	0.007
3	AND	1	0	0.01	0	0.01	1.0	0.1	5	0.72	0.85	0.58	0.67	0.70	4.236	0.310
3	AND	1	1	0.01	0	0.01	10000.0	0.1	4	0.71	0.88	0.54	0.66	0.69	6.201	0.433
3	OR	0	0	0	0	0.2	1.0	0.1	10	0.50	1.00	0.00	0.50	0.00	0.488	0.013
3	OR	0	1	0	0	0.5	0.001	0.1	4	0.50	1.00	0.00	0.50	0.00	0.377	0.008
3	OR	1	0	0.01	0	0.01	10000.0	0.1	4	0.64	0.99	0.29	0.58	0.54	4.834	0.432
3	OR	1	1	0.01	0	0.01	10000.0	0.1	4	0.55	0.99	0.11	0.53	0.33	3.531	0.549
3	UNI$_0$	0	0	0	0	0.01	10000.0	0.1	3	0.39	0.70	0.09	0.43	0.24	0.337	0.008
3	UNI$_0$	0	1	0	0	0.01	0.01	0.1	2	0.41	0.71	0.11	0.44	0.28	0.297	0.007
3	UNI$_0$	1	0	0.01	0	0.01	1.0	0.1	5	0.72	0.85	0.58	0.67	0.70	3.763	0.743
3	UNI$_0$	1	1	0.01	0	0.01	10000.0	0.1	4	0.71	0.88	0.54	0.66	0.69	4.450	0.694
3	UNI$_1$	0	0	0	0	0.01	10.0	0.1	5	0.48	0.88	0.07	0.49	0.25	0.491	0.012
3	UNI$_1$	0	1	0	0	0.01	0.001	0.1	5	0.46	0.87	0.06	0.48	0.23	0.411	0.010
3	UNI$_1$	1	0	0.01	0	0.01	10000.0	0.1	4	0.76	0.93	0.59	0.69	0.74	8.064	0.430
3	UNI$_1$	1	1	0.01	0	0.01	10000.0	0.1	4	0.73	0.93	0.52	0.66	0.70	5.040	0.509
3	UNI$_2$	0	0	0	0	0.01	10.0	0.1	5	0.48	0.88	0.07	0.49	0.25	0.436	0.010
3	UNI$_2$	0	1	0	0	0.01	0.001	0.1	5	0.46	0.87	0.06	0.48	0.23	0.537	0.014
3	UNI$_2$	1	0	0.01	0	0.01	10000.0	0.1	4	0.76	0.93	0.59	0.69	0.74	3.428	1.247
3	UNI$_2$	1	1	0.01	0	0.01	10000.0	0.1	4	0.73	0.93	0.52	0.66	0.70	4.835	0.708
4	AND	0	0	0	0	0.01	0.01	0.1	2	0.39	0.70	0.08	0.43	0.24	0.265	0.007
4	AND	0	1	0	0	0.01	10.0	0.1	5	0.42	0.71	0.13	0.45	0.31	0.404	0.010
4	AND	1	0	0.01	0	0.01	10000.0	0.1	4	0.80	0.84	0.76	0.78	0.80	3.374	0.279
4	AND	1	1	0.01	0	0.01	10000.0	0.1	4	0.67	0.85	0.48	0.62	0.64	4.070	0.574
4	OR	0	0	0	0	0.01	1000.0	0.1	3	0.50	1.00	0.01	0.50	0.08	0.279	0.008
4	OR	0	1	0	0	0.01	10.0	0.1	5	0.50	1.00	0.01	0.50	0.08	0.437	0.010
4	OR	1	0	0.01	0	0.01	1.0	0.1	5	0.60	0.99	0.21	0.55	0.45	3.145	0.471
4	OR	1	1	0.01	0	0.01	10000.0	0.1	4	0.57	0.99	0.15	0.54	0.39	3.903	0.394
4	UNI$_0$	0	0	0	0	0.01	0.01	0.1	2	0.39	0.70	0.08	0.43	0.24	0.241	0.005
4	UNI$_0$	0	1	0	0	0.01	10.0	0.1	5	0.42	0.71	0.13	0.45	0.31	0.272	0.008
4	UNI$_0$	1	0	0.01	0	0.01	10000.0	0.1	4	0.80	0.84	0.76	0.78	0.80	3.355	0.379
4	UNI$_0$	1	1	0.01	0	0.01	10000.0	0.1	4	0.67	0.85	0.48	0.62	0.64	4.606	0.695
4	UNI$_1$	0	0	0	0	0.01	0.01	0.1	2	0.46	0.85	0.07	0.48	0.25	0.328	0.008
4	UNI$_1$	0	1	0	0	0.01	10.0	0.1	5	0.48	0.83	0.13	0.49	0.33	0.425	0.010
4	UNI$_1$	1	0	0.01	0	0.01	10000.0	0.1	4	0.71	0.96	0.47	0.64	0.67	5.069	0.422
4	UNI$_1$	1	1	0.01	0	0.01	10000.0	0.1	4	0.70	0.96	0.44	0.63	0.65	3.889	0.509
4	UNI$_2$	0	0	0	0	0.01	0.01	0.1	2	0.46	0.85	0.07	0.48	0.25	0.162	0.005
4	UNI$_2$	0	1	0	0	0.01	10.0	0.1	5	0.48	0.83	0.13	0.49	0.33	0.183	0.004
4	UNI$_2$	1	0	0.01	0	0.01	10000.0	0.1	4	0.71	0.96	0.47	0.64	0.67	3.048	0.531
4	UNI$_2$	1	1	0.01	0	0.01	10000.0	0.1	4	0.70	0.96	0.44	0.63	0.65	4.083	0.567
5	AND	0	0	0	0	0.01	10.0	0.1	5	0.37	0.61	0.13	0.41	0.28	0.405	0.010
5	AND	0	1	0	0	0.01	10.0	0.1	5	0.41	0.72	0.11	0.45	0.28	0.406	0.010

5	AND	1	0	0.01	0	0.01	1.0	0.1	5	0.76	0.85	0.66	0.72	0.75	3.399	0.526
5	AND	1	1	0.01	0	0.01	10000.0	0.1	4	0.72	0.89	0.54	0.66	0.70	3.125	0.378
5	OR	0	0	0	0	0.01	10.0	0.1	5	0.50	1.00	0.01	0.50	0.08	0.238	0.008
5	OR	0	1	0	0	0.5	0.001	0.1	4	0.50	1.00	0.00	0.50	0.00	0.242	0.006
5	OR	1	0	0.01	0	0.01	10000.0	0.1	4	0.57	0.99	0.15	0.54	0.39	7.438	0.389
5	OR	1	1	0.01	0	0.01	10000.0	0.1	4	0.55	0.99	0.10	0.52	0.32	5.155	0.574
5	UNI_0	0	0	0	0	0.01	10.0	0.1	5	0.37	0.61	0.13	0.41	0.28	0.405	0.010
5	UNI_0	0	1	0	0	0.01	100.0	0.1	3	0.42	0.73	0.11	0.45	0.28	0.458	0.011
5	UNI_0	1	0	0.01	0	0.01	1.0	0.1	5	0.76	0.85	0.66	0.72	0.75	4.777	0.308
5	UNI_0	1	1	0.01	0	0.01	10000.0	0.1	4	0.72	0.89	0.54	0.66	0.70	4.921	0.613
5	UNI_1	0	0	0	0	0.01	10.0	0.1	5	0.45	0.77	0.13	0.47	0.31	0.445	0.010
5	UNI_1	0	1	0	0	0.01	10.0	0.1	5	0.47	0.87	0.06	0.48	0.23	0.450	0.010
5	UNI_1	1	0	0.01	0	0.01	10000.0	0.1	4	0.72	0.94	0.49	0.65	0.68	3.237	0.267
5	UNI_1	1	1	0.01	0	0.01	10000.0	0.1	4	0.71	0.95	0.48	0.65	0.67	2.701	0.398
5	UNI_2	0	0	0	0	0.01	10.0	0.1	5	0.45	0.77	0.13	0.47	0.31	0.366	0.008
5	UNI_2	0	1	0	0	0.01	10.0	0.1	5	0.47	0.87	0.06	0.48	0.23	0.250	0.008
5	UNI_2	1	0	0.01	0	0.01	10000.0	0.1	4	0.72	0.94	0.49	0.65	0.68	4.346	0.480
5	UNI_2	1	1	0.01	0	0.01	10000.0	0.1	4	0.71	0.95	0.48	0.65	0.67	4.713	0.766
6	AND	0	0	0	0	0.01	10.0	0.1	5	0.40	0.71	0.10	0.44	0.26	1.075	0.010
6	AND	0	1	0	0	0.01	0.01	0.1	2	0.41	0.71	0.11	0.44	0.28	1.071	0.007
6	AND	1	0	0.01	0	0.01	1.0	0.1	5	0.72	0.85	0.58	0.67	0.70	3.422	0.291
6	AND	1	1	0.01	0	0.01	10000.0	0.1	4	0.68	0.88	0.47	0.62	0.64	4.436	0.562
6	OR	0	0	0	0	0.1	100.0	0.1	10	0.50	1.00	0.00	0.50	0.00	1.108	0.010
6	OR	0	1	0	0	0.5	0.001	0.1	4	0.50	1.00	0.00	0.50	0.00	0.930	0.007
6	OR	1	0	0.01	0	0.01	10000.0	0.1	4	0.62	0.99	0.24	0.57	0.49	5.478	0.426
6	OR	1	1	0.01	0	0.01	10000.0	0.1	4	0.54	0.99	0.10	0.52	0.31	5.299	0.640
6	UNI_0	0	0	0	0	0.01	10.0	0.1	5	0.40	0.71	0.10	0.44	0.26	1.113	0.010
6	UNI_0	0	1	0	0	0.01	0.01	0.1	2	0.41	0.71	0.11	0.44	0.28	0.884	0.008
6	UNI_0	1	0	0.01	0	0.01	1.0	0.1	5	0.72	0.85	0.58	0.67	0.70	4.194	0.389
6	UNI_0	1	1	0.01	0	0.01	10000.0	0.1	4	0.68	0.88	0.47	0.62	0.64	4.774	0.723
6	UNI_1	0	0	0	0	0.01	0.001	0.1	5	0.48	0.88	0.07	0.49	0.25	0.622	0.010
6	UNI_1	0	1	0	0	0.01	0.001	0.1	5	0.46	0.87	0.06	0.48	0.23	1.232	0.010
6	UNI_1	1	0	0.01	0	0.01	10000.0	0.1	4	0.77	0.93	0.61	0.71	0.75	5.827	0.387
6	UNI_1	1	1	0.01	0	0.01	10000.0	0.1	4	0.70	0.94	0.45	0.63	0.65	5.989	0.522
6	UNI_2	0	0	0	0	0.01	0.001	0.1	5	0.48	0.88	0.07	0.49	0.25	1.009	0.008
6	UNI_2	0	1	0	0	0.01	0.001	0.1	5	0.46	0.87	0.06	0.48	0.23	0.680	0.008
6	UNI_2	1	0	0.01	0	0.01	10000.0	0.1	4	0.77	0.93	0.61	0.71	0.75	4.458	0.542
6	UNI_2	1	1	0.01	0	0.01	10000.0	0.1	4	0.70	0.94	0.45	0.63	0.65	5.673	0.648
7	AND	0	0	0	3	0.01	1000.0	0.1	10	0.41	0.70	0.11	0.44	0.28	0.911	0.014
7	AND	0	1	0	4	0.01	10.0	0.1	3	0.43	0.73	0.13	0.45	0.30	0.832	0.004
7	AND	1	0	0.01	7	0.01	0.01	0.1	3	0.77	0.85	0.70	0.74	0.77	3.666	0.395
7	AND	1	1	0.01	6	0.01	1.0	0.1	2	0.76	0.86	0.66	0.72	0.75	4.915	0.835
7	OR	0	0	0	2	0.01	0.001	0.1	10	0.51	1.00	0.01	0.50	0.11	1.197	0.014
7	OR	0	1	0	2	0.01	0.001	0.1	10	0.50	1.00	0.01	0.50	0.08	1.318	0.018
7	OR	1	0	0.01	6	0.01	1.0	0.1	2	0.56	1.00	0.12	0.53	0.35	3.160	0.479
7	OR	1	1	0.01	6	0.01	0.01	0.1	2	0.59	0.99	0.19	0.55	0.43	3.651	0.389
7	UNI_0	0	0	0	3	0.01	1000.0	0.1	10	0.41	0.70	0.11	0.44	0.28	0.732	0.007
7	UNI_0	0	1	0	9	0.01	10.0	0.1	3	0.43	0.73	0.13	0.45	0.30	1.440	0.007
7	UNI_0	1	0	0.01	3	0.01	0.001	0.1	2	0.72	0.84	0.60	0.68	0.71	4.479	0.414
7	UNI_0	1	1	0.01	3	0.01	0.001	0.1	2	0.70	0.77	0.62	0.67	0.69	3.966	0.739
7	UNI_1	0	0	0	9	0.01	0.001	0.1	5	0.47	0.84	0.11	0.48	0.30	1.049	0.022
7	UNI_1	0	1	0	1	0.01	100.0	0.1	3	0.48	0.85	0.12	0.49	0.32	0.849	0.007
7	UNI_1	1	0	0.01	7	0.01	1000.0	0.1	2	0.77	0.95	0.59	0.70	0.75	2.866	0.397
7	UNI_1	1	1	0.01	6	0.01	0.01	0.1	2	0.78	0.94	0.63	0.72	0.77	3.867	0.755
7	UNI_2	0	0	0	9	0.01	0.001	0.1	5	0.47	0.84	0.11	0.48	0.30	0.473	0.008
7	UNI_2	0	1	0	10	0.01	1.0	0.1	3	0.48	0.85	0.12	0.49	0.32	0.874	0.009
7	UNI_2	1	0	0.01	7	0.01	1000.0	0.1	2	0.77	0.95	0.59	0.70	0.75	3.506	0.476
7	UNI_2	1	1	0.01	6	0.01	0.01	0.1	2	0.78	0.94	0.63	0.72	0.77	4.260	0.382
8	AND	0	0	0	9	0.01	0.001	0.1	5	0.40	0.70	0.09	0.44	0.25	9.557	0.013
8	AND	0	1	0	3	0.01	0.1	0.1	20	0.42	0.73	0.10	0.45	0.28	10.318	0.029
8	AND	1	0	0.01	6	0.01	0.1	0.1	3	0.74	0.85	0.64	0.70	0.74	11.719	2.014
8	AND	1	1	0.01	3	0.01	0.001	0.1	2	0.75	0.85	0.64	0.70	0.74	14.674	0.803
8	OR	0	0	0	3	0.01	0.1	0.1	20	0.50	1.00	0.01	0.50	0.08	9.020	0.057
8	OR	0	1	0	3	0.01	1000.0	0.1	10	0.50	1.00	0.00	0.50	0.00	9.580	0.015
8	OR	1	0	0.01	5	0.01	0.1	0.1	4	0.60	0.99	0.21	0.56	0.46	13.260	0.526
8	OR	1	1	0.01	3	0.01	0.001	0.1	2	0.61	0.98	0.24	0.56	0.49	13.887	1.648
8	UNI_0	0	0	0	9	0.01	0.001	0.1	5	0.40	0.70	0.09	0.44	0.25	10.592	0.030
8	UNI_0	0	1	0	3	0.01	0.1	0.1	20	0.42	0.73	0.10	0.45	0.28	9.406	0.012

8	UNI$_0$	1	0	0.01	6	0.01	0.1	0.1	3	0.74	0.85	0.64	0.70	0.74	13.447	0.931
8	UNI$_0$	1	1	0.01	3	0.01	0.001	0.1	2	0.75	0.85	0.64	0.70	0.74	12.984	1.407
8	UNI$_1$	0	0	0	8	0.01	10000.0	0.1	4	0.47	0.85	0.09	0.48	0.28	10.258	0.005
8	UNI$_1$	0	1	0	3	0.01	0.1	0.1	20	0.48	0.85	0.10	0.49	0.29	9.580	0.012
8	UNI$_1$	1	0	0.01	7	0.01	1000.0	0.1	2	0.80	0.95	0.65	0.73	0.78	13.570	1.313
8	UNI$_1$	1	1	0.01	3	0.01	0.001	0.1	2	0.77	0.93	0.62	0.71	0.76	14.704	0.849
8	UNI$_2$	0	0	0	9	0.01	0.001	0.1	5	0.47	0.85	0.09	0.48	0.28	10.036	0.027
8	UNI$_2$	0	1	0	3	0.01	0.1	0.1	20	0.48	0.85	0.10	0.49	0.29	9.755	0.019
8	UNI$_2$	1	0	0.01	7	0.01	1000.0	0.1	2	0.8	0.95	0.65	0.73	0.78	13.283	0.926
8	UNI$_2$	1	1	0.01	3	0.01	0.001	0.1	2	0.77	0.93	0.62	0.71	0.76	11.677	1.333
9	AND	0	0	0	3	0.01	0.1	0.1	20	0.40	0.67	0.12	0.43	0.29	7.986	0.023
9	AND	0	1	0	3	0.01	0.1	0.1	20	0.43	0.73	0.13	0.45	0.30	7.872	0.046
9	AND	1	0	0.01	6	0.01	0.1	0.1	3	0.79	0.84	0.74	0.77	0.79	11.806	0.876
9	AND	1	1	0.01	7	0.01	0.1	0.1	2	0.79	0.89	0.68	0.74	0.78	11.878	1.488
9	OR	0	0	0	5	0.01	0.1	0.1	5	0.51	1.00	0.01	0.50	0.11	7.389	0.020
9	OR	0	1	0	3	0.01	1000.0	0.1	10	0.50	1.00	0.00	0.50	0.00	7.602	0.011
9	OR	1	0	0.01	3	0.01	100.0	0.1	4	0.60	0.99	0.20	0.55	0.45	10.969	0.518
9	OR	1	1	0.01	7	0.01	1000.0	0.1	2	0.62	0.98	0.26	0.57	0.51	13.279	1.499
9	UNI$_0$	0	0	0	3	0.01	0.1	0.1	20	0.40	0.67	0.12	0.43	0.29	8.080	0.023
9	UNI$_0$	0	1	0	3	0.01	0.1	0.1	20	0.43	0.73	0.13	0.45	0.30	8.194	0.024
9	UNI$_0$	1	0	0.01	6	0.01	0.1	0.1	3	0.79	0.84	0.74	0.77	0.79	11.054	0.561
9	UNI$_0$	1	1	0.01	7	0.01	0.1	0.1	2	0.79	0.89	0.68	0.74	0.78	12.141	1.754
9	UNI$_1$	0	0	0	3	0.01	0.1	0.1	20	0.48	0.84	0.12	0.49	0.32	8.237	0.024
9	UNI$_1$	0	1	0	3	0.01	0.1	0.1	20	0.49	0.85	0.12	0.49	0.32	8.035	0.027
9	UNI$_1$	1	0	0.01	7	0.01	1000.0	0.1	2	0.80	0.95	0.65	0.73	0.78	12.685	1.258
9	UNI$_1$	1	1	0.01	7	0.01	0.1	0.1	2	0.80	0.96	0.63	0.72	0.78	10.947	1.584
9	UNI$_2$	0	0	0	3	0.01	0.1	0.1	20	0.48	0.84	0.12	0.49	0.32	8.228	0.015
9	UNI$_2$	0	1	0	3	0.01	0.1	0.1	20	0.49	0.85	0.12	0.49	0.32	7.890	0.012
9	UNI$_2$	1	0	0.01	7	0.01	1000.0	0.1	2	0.80	0.95	0.65	0.73	0.78	11.907	0.638
9	UNI$_2$	1	1	0.01	7	0.01	0.1	0.1	2	0.80	0.96	0.63	0.72	0.78	13.457	0.751

Table 2: Full results for MS-SVDD experiments.
